# Real‐World Experience of Sustained‐Release Fampridine in Chinese Patients With Multiple Sclerosis: A Case Series on Walking Impairment and Fatigue

**DOI:** 10.1155/bn/6426255

**Published:** 2025-12-25

**Authors:** Han Wang, Wenting Zhang, Fengjun Wang, Qin Wang, Tao Yang, Runze Zhao, RongRong Wang, Antonio Carrillo-Vico, Guillermo Izquierdo, Yu Zhang, Xiongfei Zhao, Guoxun Zhang

**Affiliations:** ^1^ Department of Neurology, Yan’an University Medical College No. 3 Affiliated Hospital, Xianyang, Shaanxi, China; ^2^ Department of Gastroenterology and Hepatology, Xijing Hospital, Air Force Medical University, Xi’an, Shaanxi, China, fmmu.edu.cn; ^3^ College of International Cooperation, Xi’an International University, Xi’an, Shaanxi, China, xaiu.edu.cn; ^4^ Department of Endoscopy Center, The Sixth Affiliated Hospital of Sun Yat-sen University, Guangzhou, Guangdong, China, sysu.edu.cn; ^5^ Department of Ophthalmology, Eye Institute of PLA, Xijing Hospital, Air Force Medical University, Xi’an, Shaanxi, China, fmmu.edu.cn; ^6^ Department of Neurology, Instituto de Biomedicina de Sevilla, IBiS (Universidad de Sevilla, HUVR, Junta de Andalucía, CSIC), Sevilla, Andalusia, Spain, ibis-sevilla.es; ^7^ Department of Neurology, Hospital Vithas Nisa Sevilla, Sevilla, Andalusia, Spain; ^8^ Department of Medical Affairs, ER-Biomed (Shanghai) Pharmaceutical Technology Co., Ltd, Shanghai, China

**Keywords:** fatigue, multiple sclerosis, sustained-release fampridine tablets, walking impairment

## Abstract

**Objectives:**

To investigate the efficacy of sustained‐release fampridine tablets (fampridine‐SR) on walking impairment and fatigue on Chinese patients with multiple sclerosis (MS).

**Methods:**

All patients (*n* = 12) had the baseline Expanded Disability Status Scale (EDSS) at 4–7 and orally administered fampridine‐SR at 10 mg twice per day for at least 12 weeks. All patients were assessed using EDSS, Timed 25‐Foot Walk (T25FW), the 12‐item Multiple Sclerosis Walking Scale (MSWS‐12), and Modified Fatigue Impact Scale (MFIS) at baseline, day 1, week 1, 2, 4, 8, and 12.

**Results:**

The baseline EDSS score was 4.67 ± 0.36. Fampridine‐SR significantly decreased the EDSS score by 0.63 ± 0.20 (*p* = 0.011) after 12‐week treatment. T25FW was changed at day 1 and week 1 by − 12.73 ± 3.03% and − 14.20 ± 4.36% (*p* < 0.011), respectively. The statistically and clinically significant improvement of MSWS‐12 was observed since week 1. The total, cognitive subscale, physical subscale, and psychosocial subscale of MFIS were significantly reduced in Chinese patients with MS.

**Conclusion:**

Fampridine‐SR was a fast‐acting oral potassium channel blocker on improving walking ability of MS as early as day 1. It demonstrated the positive effects on walking impairment and fatigue, including the physical, cognitive, and psychosocial subscales of MFIS, in Chinese patients with MS.

## 1. Introduction

Multiple sclerosis (MS) is a heterogenous demyelination disease of central nervous system. Walking impairment is one of the most common symptoms in patients with MS, which has negatively affected patients’ quality of life [1]. It is reported that more than 93% MS patients have reported walking problems within 10 years of diagnosis [2]. In addition, walking impairment placed a heavy burden to independence, employment, psychosocial health, caregiving, and economy not only to MS patients but also to their family [3, 4]. Many efforts have been made to improve the walking function of patients with MS, including rehabilitation, physical therapy, drug development, and walking aids [5–7].

Sustained‐release fampridine tablet (fampridine‐SR) is an oral potassium channel blocker which prevent K^+^ efflux to restore conduction in focally demyelinated axons [8]. Improvement of walking function by fampridine‐SR has been proved by MS‐F203 and MS‐F204 [6, 9]. The indication was approved as early as 2010 by U.S. Food and Drug Administration (FDA). Real‐world studies have shown that fampridine‐SR could improve fatigue, cognition, and depression [10, 11], improving quality of life in patients with MS.

Fampridine‐SR was approved to improve walking function in MS patients with walking impairment (EDSS 4‐7) by China National Medical Products Administration (NMPA) in 2021 [12]. However, because fampridine‐SR was approved through an accelerated process with the clinical trial waiver, there is a lack of efficacy and safety data of fampridine‐SR on Chinese patients with MS. Therefore, this case series analysis aimed to investigate the efficacy of fampridine‐SR on walking impairment, fatigue, cognition, and psychosocial ability patients, providing the real‐world experience of fampridine‐SR on Chinese patients with MS.

## 2. Methods

### 2.1. Patients

This is a single‐center case series study. Twelve patients have been included from March 2023 to November 2023 in Yan’an University Medical College No. Three Affiliated Hospital, Xianyang. All patients were clinically defined MS using 2017 revisions of the McDonald criteria. The included patients should be at ≥ 18 years old. The baseline Expanded Disability Status Scale (EDSS) should be 4–7. All patients were prescribed fampridine‐SR orally at 10 mg twice per day for at least 12 weeks. All patients were required to complete the walking and fatigue evaluation described below at baseline, day 1, week 1, week 2, week 4, week 8, and week 12.

This study was approved by the Ethics Committee of Yan’an University Medical College No. Three Affiliated Hospital (Approval No. YDXY‐KY‐2022‐012). The study was conducted according to the principles of the Declaration of Helsinki [13]. All patients have signed the informed consent.

### 2.2. Evaluation of Walking Function and Fatigue

EDSS is the gold standard for assessing disability of MS [14] and was used to evaluate walking function in this study. The clinically meaningful EDSS worsening was defined as an increased score of ≥ 1.0 point while the clinically meaningful EDSS improvement was defined as a reduced score of ≥ 1.0 point [15].

The walking speed was measured using Timed 25‐Foot Walk (T25FW). T25FW should be done twice for each evaluation, and the average value was used for analysis. The maximum of 5‐min rest was allowed between tests. The clinically meaningful change of walking speed was defined as ≥ 20% change of the T25FW test [16] at least one visit during the treatment.

The 12‐item Multiple Sclerosis Walking Scale (MSWS‐12) is a widely used self‐reported questionnaires for evaluating walking ability (J. C. [17]), which was also used in this study to evaluated patient‐reported walking ability. The clinically meaningful improvement was defined as the reduction of ≥ 8 points in MSWS‐12 score [18] at least one visit during the treatment.

Fatigue was assessed using Modified Fatigue Impact Scale (MFIS). All patients should fill in the MFIS at each visit. The three subscales (physical, cognitive, and psychosocial) of MFIS were also analyzed in this study. The clinically meaningful change of fatigue was defined as the change of MFIS of ≥ 4 points in MFIS [19] at least one visit during the treatment.

### 2.3. Statistical Analysis

All results were presented as mean ± standard error of mean (SEM). The statistical analysis was performed using SPSS software 26.0 (IBM SPSS, United States). Difference between two groups was analyzed using Student *t* test under abnormal distribution. A *p* value less than 0.05 was defined as statistical significance.

## 3. Results

### 3.1. Patient Characteristics

Twelve MS patients were included in this study (Table 1). Ten of them were female and two were male. The patient baseline characteristics are shown in Table 1. The mean age was 35.50 ± 1.82 years old. The mean disease duration was 5.67 ± 1.10 years with the longest duration of 12 years and the shortest duration of 1 year. The mean baseline EDSS score was 4.67 ± 0.36. Nine of them had the baseline EDSS score at 4.0. All patients have received fampridine‐SR at 10 mg twice per day for at least 12 weeks and completed seven visits as required.

**Table 1:** Patients baseline characteristics.

**Item**	**n** = 12
Age, years	35.50 ± 1.82
Sex	10 (83.33%)
Female	10 (83.33%)
Male	2 (16.67)
Disease duration, years	5.67 ± 1.10
MS subtype	
RRMS	10 (83.33)
SPMS	2 (16.67)
DMT treatment	
Yes	6 (50.00)
No	6 (50.00)
EDSS	4.67 ± 0.36
T25FW	15.05 ± 4.15
MSWS‐12	64.58 ± 6.53
MFIS	44.00 ± 3.81

*Note:* All data were shown as mean ± SEM or number (percentage, %).

Abbreviations: DMT, disease‐modifying therapy; EDSS, Expanded Disability Status Scale; MFIS, Modified Fatigue Impact Scale; MS: multiple sclerosis; MSWS12, the 12‐item Multiple Sclerosis Walking Scale; T25FW, Timed 25‐Foot Walk.

### 3.2. Improvement of Walking Function After Fampridine‐SR Treatment

Walking function was measured using three different measures (Table 2). The changes of EDSS score demonstrated a statistically significant difference only at week 12 compared to baseline. Four (33.33%) of them showed the clinically meaningful EDSS improvement, and no patient had the EDSS worsening. The statistically significant difference was only observed at day 1 and week 1 when evaluated by T25FW, although a trend of improvement was shown in all visits. The average changes of T25FW were less than 20%, which were not clinically meaningful. MSWS‐12 assessment was significantly improved from week 1 to week 12 compared to baseline. The MSWS‐12 score was reduced by 9.50 ± 4.37 at week 1 and the higher reductions were observed in the following visits, which were clinically meaningful improvement [18].

**Table 2:** Improvement of walking function after fampridine‐SR treatment.

**Change from baseline**		**Day 1**	**Week 1**	**Week 2**	**Week 4**	**Week 8**	**Week 12**
EDSS	Score	0.00 ± 0.00	0.00 ± 0.04	− 0.04 ± 0.07	− 0.17 ± 0.09	− 0.33 ± 0.19	− 0.63 ± 0.20
	*p* value	—	1.000	0.581	0.152	0.090	0.011

T25FW	Percentage (%)	− 12.73 ± 3.03	− 14.20 ± 4.36	− 10.98 ± 10.40	− 10.70 ± 9.00	− 7.83 ± 10.42	− 8.44 ± 12.69
	*p* value	0.000	0.004	0.303	0.247	0.460	0.528

MSWS‐12	Score	− 1.75 ± 1.45	− 9.50 ± 4.37	− 12.08 ± 5.15	− 17.75 ± 5.26	− 15.42 ± 3.61	− 16.83 ± 3.63
	*p* value	0.239	0.041	0.028	0.003	0.000	0.001

*Note:* Data were shown as mean ± SEM. *p* < 0.05 was defined as statistical significance compared to baseline.

### 3.3. Improvement of Fatigue After Fampridine‐SR Treatment

The standard 21‐item version of MFIS was filled by the patients, and the total and three subscales (physical, cognitive, and psychosocial) were analyzed in this study (Figure 1). The total and psychosocial subscale of MFIS score was significantly improved from week 1 to week 12 (Figures 1a,d). The improvement of physical subscale was observed from week 1 to week 8, and no significant improvement was observed at week 12 (Figure 1b). The improvement of cognitive subscale was observed from week 2 to week 12 (Figure 1c).

Figure 1Improvement of fampridine on fatigue in Chinese MS patients. (a) Total, (b) physical subscale, (c) cognitive subscale, and (d) psychosocial subscale.

**Figure:**
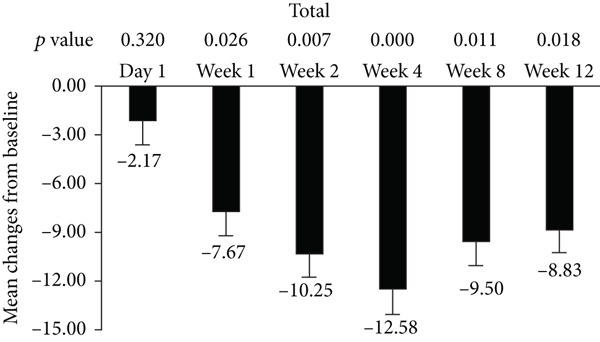
(a)

**Figure:**
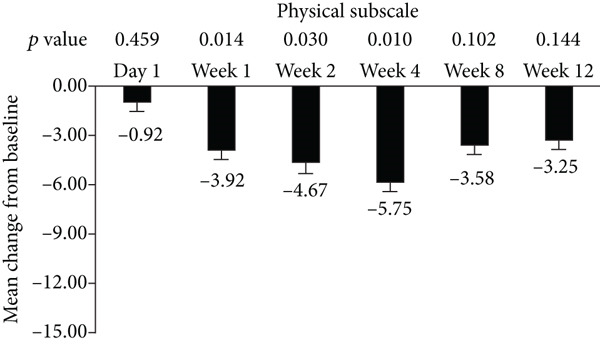
(b)

**Figure:**
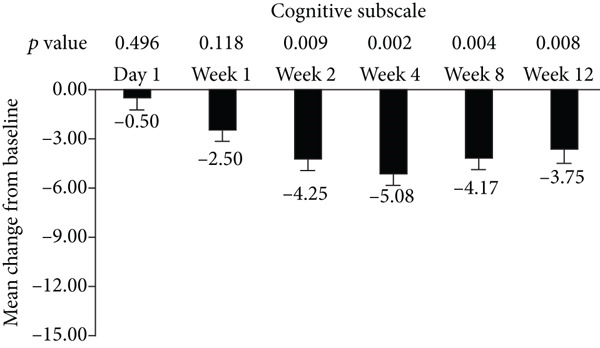
(c)

**Figure:**
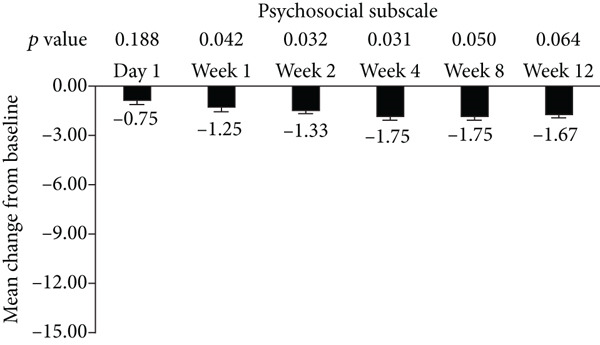
(d)

### 3.4. Treatment Response to Fampridine‐SR Using Different Assessments

A detailed treatment response has been analyzed in each patient (Table 2). Among the 12 patients, 4 (33.33%) demonstrated EDSS improvement. No patients had EDSS worsening. Nine patients (75%) showed the improvement on walking speed (T25FW). Ten patients (83.33%) experienced the clinically meaningful improvement on MSWS‐12 results. Ten patients (83.33%) reported the improvement on fatigue. Only one patient had no improvement on all the assessments. Therefore, the multidimensional assessments should be done when evaluating the response to fampridine‐SR.

## 4. Discussion

Fampridine‐SR has been approved by FDA in 2010 and conditionally approved by European Medicines Agency in 2011 [12]. Therefore, a huge amount of real‐world data has been accumulated in the past 10 years. However, there were only 2 years since it was approved in China. It was in a great need to fill the data gap in Chinese patients with MS. For the first time, we proved the effectiveness of fampridine‐SR on walking impairment and fatigue in Chinese patients with MS, which provides the evidence for Chinese neurologists to treat MS patients with walking impairment and fatigue confidently.

EDSS and T25FW are the gold standard for assessing walking impairment of MS [14]. Improvement of motor function by fampridine was firstly reported in 1987 [20]. It took longer time for EDSS improvement compared to T25W and MSWS‐12. The statistically significant change of EDSS was only observed at week 12 while the improvement of MSWS‐12 was observed as early as week 1. The significant changes of T25FW were observed at day 1 and week 1 but diminished at weeks 4, 8, and 12. Different measurements indicated different response and effective time [21, 22]. Meanwhile, individual variations were observed when using different measurements. At least two patients showed no improvement to each single assessment (EDSS, T25FW, MSWS‐12, or MFIS). But only one patient demonstrated no response to all assessments. In accordance with the published findings, the composite response criteria demonstrated a higher rate of responders compared to the single assessment [21]. Therefore, a multidimensional evaluation is needed to assess the efficacy and response of fampridine in Chinese patients with MS.

It is interesting to note that the improvement of T25FW could be observed since day 1. Similar results were reported by Stefoski et al. in 1991 that the improvement of gait function occurred within 1–3 h post dose [23, 24]. These results implied that fampridine was a fast‐acting oral K^+^ blocker on improving walking ability of MS. It should be mentioned that the two patients with secondary progressive MS showed no MSWS‐12 improvement and T25FW worsening (data no shown), indicating that patients with MS should be treated with fampridine‐SR early to maximize the clinical benefit on walking impairment.

In addition to walking function, fatigue is also one of most common MS symptoms with the prevalence of 36.5%–78.0% which negatively influences patients’ quality of life [25]. Improvement of fatigue by fampridine‐SR have been proved by previous studies [21, 26]. In accordance with these published results, fampridine‐SR reduced the total scores of MFIS since Week 1 in Chinese patients with MS. Cognitive functions have got more and more attentions in MS in the past years [27, 28]. The cognitive subscale was significantly reduced after 2‐week fampridine treatment in Chinese patients with MS, which were consistent with the previous findings in non‐Chinese patients with MS [10, 29]. Physical and psychosocial subscales were also significant improved, suggesting that fampridine could improve the multiple symptoms induced by demyelination of CNS in Chinese patients with MS.

There were several limitations of this study: (1) The sample size was small because MS is defined as a rare disease, and it is difficult to recruit patients within a short time; a multicenter, large clinical observation should be conducted to obtain the solid evidence to guide clinical use of fampridine‐SR in patients with MS. (2) Only walking function and fatigue was evaluated in this study and more other symptoms should be evaluated in future, such as upper limbs, visual function, and other nonwalking functions [21, 30] to generate more evidence to maximize clinical benefit in Chinese patients with MS. (3) The patients were only followed up for 12 weeks, indicating that there is lack of long‐term data of fampridine‐SR in Chinese patients with MS, so the long‐term efficacy of fampridine should be explored in Chinese patients with MS in future.

## 5. Conclusion

To our knowledge, this was the first study reporting the efficacy of fampridine‐SR in Chinese patients with MS. Fampridine‐SR demonstrated the positive effects on walking impairment and fatigue, including the physical, cognitive, and psychosocial subscales of MFIS, in Chinese patients with MS, which provided the confidence to Chinese neurologists and patient in the management of MS symptoms. However, the large, multicenter study and a long‐term follow‐up should be conducted to validate the efficacy of fampridine in Chinese patients with MS.

## Ethics Statement

This study was approved by the Ethics Committee of Yan’an University Medical College No. Three Affiliated Hospital (Approval No. YDXY‐KY‐2022‐012). All the patients allowed personal data processing, and informed consent was obtained from all individual participants included in the study.

## Conflicts of Interest

Yu Zhang is an employee from ER‐Biomed (Shanghai). All other authors declare that there were no conflicts of interest.

## Author Contributions

Han Wang: formal analysis, validation, writing—review and editing. Wenting Zhang: formal analysis, validation, writing—review and editing. Fengjun Wang: formal analysis, validation, writing—review and editing. Antonio Carrillo‐Vico: conceptualization, formal analysis, validation. G. Izquierdo: conceptualization, formal analysis, validation. Yu Zhang: literature review, validation, writing—review and editing. Xiongfei Zhao: conceptualization, validation, writing—review and editing. Guoxun Zhang: conceptualization, project administration, supervision, writing—review and editing. Tao Yang, Runze Zhao and Rongrong Wang assist in data collection and provide advice. Prof. Guoxun Zhang is lead contact. Han Wang, Wenting Zhang, and Fengjun Wang equally contributed to this work and are regarded as co‐first authors.

## Funding

This study is supported by the Yan’an University Medical College No. Three Affiliated Hospital, 2025KY016.

## Data Availability

The data that support the findings of this study are available from the corresponding author upon reasonable request.
